# Cross-Leg as Salvage Procedure after Free Flaps Transfer Failure: A Case Report

**DOI:** 10.1155/2012/205029

**Published:** 2012-03-29

**Authors:** F. Contedini, L. Negosanti, E. Fabbri, V. Pinto, B. Tavaniello, R. Sgarzani, R. Cipriani

**Affiliations:** Plastic Surgery Department, S.Orsola-Malpighi Hospital, 40138 Bologna, Italy

## Abstract

Posttraumatic wounds of the lower leg with soft tissue defects and exposed fractures are a reconstructive challenge due to the scarce availability of local tissues and recipient vessels. Even when a free tissue transfer can be performed the risk of failure remains considerable. When a free flap is contraindicated or after a free flap failure, the cross-leg flap is still nowadays a possible option. We report a case of a male with a severe posttraumatic wound of the lower leg with exposed tibia fracture firstly treated with two consecutive latissimus dorsi muscular free flaps, failed for vascular thrombosis; the coverage was then achieved with a cross-leg flap with acceptable results.

## 1. Introduction

Reconstruction of lower leg posttraumatic wounds, especially with exposed fracture, is generally achieved with muscular or myocutaneous/fasciocutaneous local or free flaps. In some cases local tissues are so severely damaged that there are no possibilities for local flaps harvesting or free flaps transfer due to the absence of available recipient vessels [1]. Even if a free flap is possible it is well known that the possibility of failure is high due to multiple factors, related to the patient (age, diabetes and smoking habits), local factors (vessel obstruction and previous radiotherapy), or to the type of flap (type of anastomosis performed, operating time, and cold ischemia time) [2]. The cross-leg flap dates back to 1854, when it was described by Hamilton to cure a chronic ulcer and after that it was successfully used for soft tissue coverage in the distal leg, especially during second world war. After diffusion of microsurgery since 1970, pedicled cross-extremity flaps for lower limb wound coverage have been replaced by free tissue transfer, but in the aforementioned cases the cross-leg flap [3] can still be considered a simple and effective alternative.

## 2. Case Presentation

We report a case of a 32-year-old male, who sustained a severe crush trauma resulting in open fractures of right tibia and acetabulum, multiple rib fractures, and closed fracture of both forearms. The patient was firstly treated in another hospital: fractures were reduced, and right tibia was covered with a free left latissimus dorsi muscular flap. After 5 days a complete necrosis of the flap occurred, the flap was removed, and a topical negative pressure therapy was applied.

The patient was then referred to our department. He presented a wound of 30 × 12 cm on the anterior surface of right leg with exposed fracture of tibia and ischemic sufferance of the distal bone segment.

We agreed that the best option was a debridement of all nonvascularised tissues and coverage with a free muscular flap, and we planned a reconstruction with the right latissimus dorsi muscular flap.

The patient was studied preoperatively with an arteriography of the right leg, that documented an obstruction of the anterior tibial artery in its middle third and was distally perfused by a branch of the interosseus artery; the posterior tibial artery was open, and the dorsalis pedis artery was frail and thin.

Surgery consisted in a removal of cortical bone from the fracture margins and distal fibular osteotomy and a soft tissues debridement. Posterior tibial vessels were isolated through a posterior leg access. The right latissimus dorsi muscular flap was harvested, transferred to cover the wound and vascularized by end-to-end venous and end-to-side arterial anastomosis between thoracodorsal and posterior tibial vessels. The muscle was covered by a mesh split thickness skin graft.

After 24 hours we observed a flap congestion, so we took back the patient to the operating room and explored the anastomosis; the venous anastomosis appeared thrombosed. The thrombus was removed and both arterial and venous anastomosis were reperformed. After surgery the flap appeared viable.

After 48 hours a flap congestion was newly observed and anastomosis exploration revealed a thrombosis of both artery and vein. We decided for the removal of latissimus dorsi muscle flap and coverage with a cross-leg fasciocutaneous flap from contralateral leg.

The flap was designed on the posteromedial surface of the left leg, and the dimension was 12 × 10 cm.

**Figure 1:**
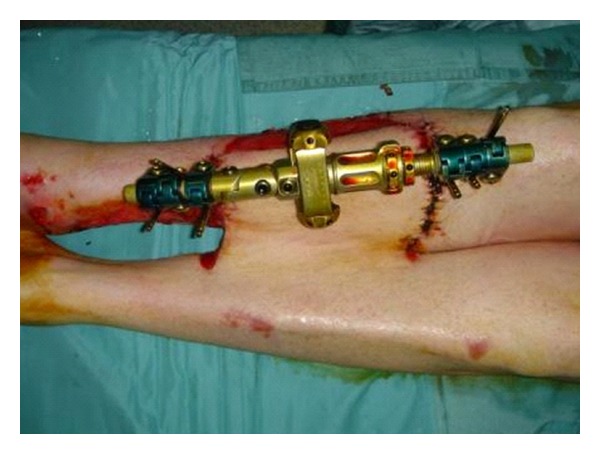
Cross-leg flap was harvested and partially sutured to the other leg to cover bone exposure.

The incision was made on the posterior, proximal, and distal side of the flap. The dissection proceeded in a subfascial plane from the posterior side to the medial one. The flap was left attached on the anterior edge, and the posterior one was sutured to the medial aspect of the bone exposure on the right leg. The other two sides were partially sutured to the wound borders (Figure 1). The remaining wound on the right leg, the under free surface of the flap, and the donor site were covered with a nonadhesive dressing. The patient was positioned using a custom-made valve for the two legs together. Moreover he presented an external fixator applied before the first surgery in another hospital It provides the necessary strength for immobilization and overhead suspension aiding greatly in wound care, as well as for general ease of patient mobility and positioning. At the same time a better immobilization is very helpful to avoid partial or total flap necrosis.

The dressing was changed every two days, and antibiotic and antithrombotic prophylaxis therapy were administered. The postoperative X-ray of the leg demonstrated a good alignment of the fracture.

**Figure 2:**
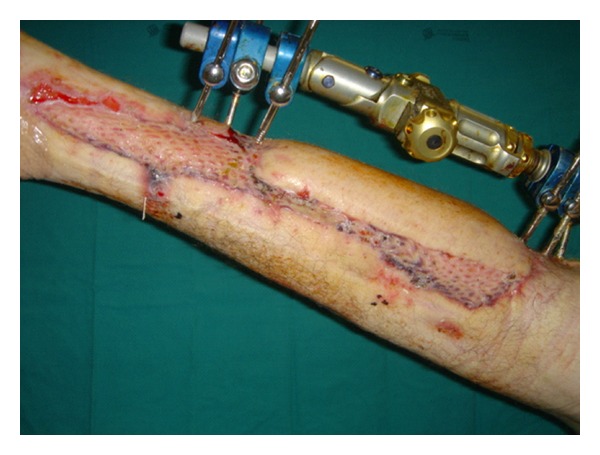
Pedicle resection after 1 month; a meshed split-thickness skin graft was used to cover remaining wound.

**Figure 3:**
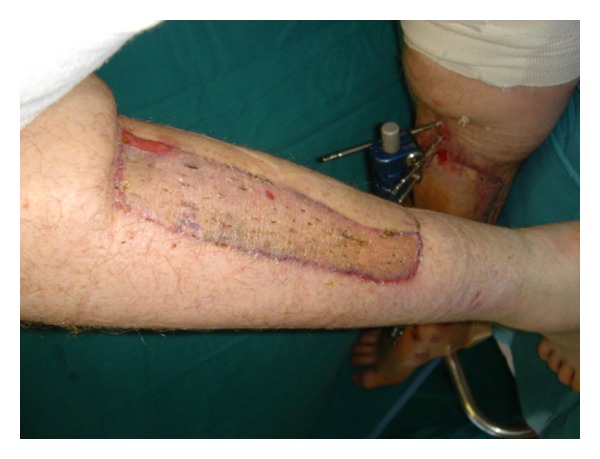
Donor site was covered with split-thickness skin graft.

After one month the flap was separated from the donor site. The donor area and the remaining wound on the right leg were covered with a skin graft (Figures 2 and 3). No complications were observed in the postoperative period and the patient was discharged after 6 days.

**Figure 4:**
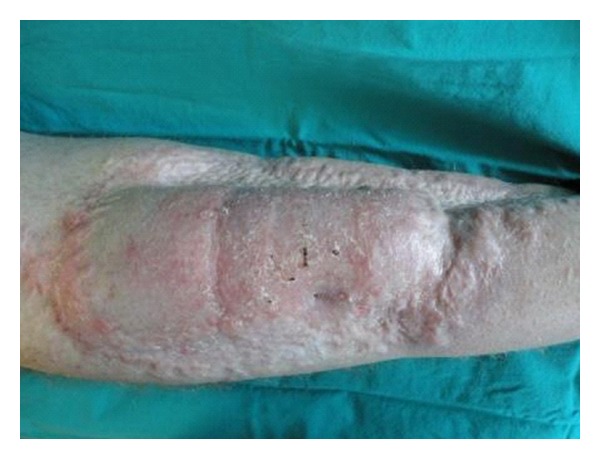
Results at 6 months of followup.

At the 6-month followup we assessed an overall satisfactory result. No neurological problems were reported, except for dysesthesia of the area surgically treated. The patient can walk, and the X-ray demonstrates a good recovery of the tibia fractures. The cosmetic result is acceptable (Figure 4).

## 3. Discussion

Soft tissues posttraumatic defects of the lower leg are a challenge for reconstruction, especially when bone or eventually a fracture is exposed.

The scarce availability of local tissues often contraindicates local flaps. Recipient vessels for free flaps transfer can be absent or severely damaged representing a contraindication to free flaps. Even general condition can be so poor that such an invasive procedure like free flap transfer could be relatively contraindicated. On the other hand, sometimes, vascular complication can cause free or local flap loss. In this cases the cross-leg flap can still be considered a useful salvage procedure as described by other authors [4].

Flap disposition is linked to flap connection with the other leg, that has to be preserved for 3 or 4 weeks to allow flap revascularization, and to its dimension because we had to maintain a base/height ratio sufficient to allow a safe vascularization (1 : 1; 1 : 1,5). For these reasons if it is not possible to cover the defect completely, as in presented case, it is important to cover bone exposure with the flap, while the remaining wound can be easily covered with a skin graft.

This flap allows, as seen in the reported case, a functional restoration with a cosmetic result nowadays still acceptable. Related to this we cannot consider cross-leg flap the first choice procedure for lower leg wound restoration, but certainly it is still a possible reconstructive technique especially after failure of other procedures.
